# Cohort Profile: Next Steps—the longitudinal study of people in England born in 1989–90

**DOI:** 10.1093/ije/dyae152

**Published:** 2024-11-13

**Authors:** Alison Fang-Wei Wu, Morag Henderson, Matt Brown, Tugba Adali, Richard J Silverwood, Darina Peycheva, Lisa Calderwood

**Affiliations:** Centre for Longitudinal Studies, Institute of Education, University College London, London, UK; Centre for Longitudinal Studies, Institute of Education, University College London, London, UK; Centre for Longitudinal Studies, Institute of Education, University College London, London, UK; Centre for Longitudinal Studies, Institute of Education, University College London, London, UK; Centre for Longitudinal Studies, Institute of Education, University College London, London, UK; Centre for Longitudinal Studies, Institute of Education, University College London, London, UK; Centre for Longitudinal Studies, Institute of Education, University College London, London, UK

**Keywords:** Cohort, millennial generation, multidisciplinary, inequalities, life course, England

Key Features

- Next Steps, previously known as the Longitudinal Study of Young People in England, is an ongoing, multidisciplinary longitudinal study of 16 122 people born in 1989**–**90. The most recent sweep of data took place when participants were aged 32 (2022**–**23). Data are available for 7279 respondents.
- The study follows individuals from adolescence through to adulthood and collects data on many aspects of life, allowing researchers and policy makers from different disciplines to document stability and change both across life and between generations. Next Steps includes measures of economic circumstances, family life, health behaviour, wellbeing, experiences during COVID-19 pandemic, and attitudes. Saliva samples were also collected at age 32, from which DNA was extracted and genotyped, which will enable researchers to unpack the genetic underpinnings of individual differences in social and behavioural outcomes.
- The Next Steps data are free to access and have been linked to administrative data, including health and education records, and can be linked to geospatial and environmental data.
- Detailed information is available on the Next Steps website [https://cls.ucl.ac.uk/cls-studies/next-steps/]. The data are available to researchers from the UK Data Service [http://ukdataservice.ac.uk], UK Longitudinal Linkage Collaboration [https://ukllc.ac.uk/datasets] and Office for National Statistics Secure Research Service [https://www.ons.gov.uk/aboutus/whatwedo/statistics/requestingstatistics/secureresearchservice].

## Why was the cohort set up?

Next Steps (previously known as the Longitudinal Study of Young People in England, LSYPE) is an ongoing, multidisciplinary, longitudinal study following 16 122 people who were in secondary school in England and were born in 1989–90. The Next Steps generation faced distinct socio-environmental pressure. For example: they grew up in a time of rapid technological change; faced unprecedented levels of student debt (an average of £50 000 upon graduating^1^); entered the labour market during the Great Recession, which meant increased job precarity; and have encountered a challenging housing market. These pressures may shape the unique health and social impacts on this generation.

The study was initially funded and managed by the Department for Education (formerly the Department for Education and Skills) and began in 2004. The recruited sample was in Year 9 (aged 13–14) of compulsory secondary education and was attending state-funded, private-funded independent schools or pupil referral units in England. Following the initial survey at age 14, cohort members were interviewed annually until 2010 (at age 20). The key aim of the study was to map participants’ journeys from compulsory schooling to university, training and, ultimately, entry into the labour market. The initial seven sweeps (2004 to 2010) mainly focused on the educational and early labour market experiences but also included diverse information on aspects of lives, including anti-social behaviours, health and wellbeing, family formation, and aspirations for the future.

In 2013, the study was transferred to the Centre for Longitudinal Studies (CLS), Institute of Education, University College London, and was funded by the Economic and Social Research Council. Since becoming part of the CLS family of longitudinal studies, the focus of Next Steps has broadened, and the study has collected information on many different aspects of life, including physical and educational development, economic circumstances, employment, family life, health behaviour, wellbeing, social participation, and attitudes. Next Steps is an important multidisciplinary and multidimensional resource for researchers and policy makers. This cohort profile comprehensively documents the aims, design and features of Next Steps, including the most recent sweeps of data collection, which broadens the scope of Calderwood and Sanchez’s data profile.^2^

## Who is in the cohort?

The Next Steps population consisted of Year 9 pupils in England in the state schools, independent schools and pupil referral units (i.e. an alternative education provision for pupils with neurodiverse learning styles, long-term illness, behavioural issues or waiting for mainstream school placement to become available) in February 2004. Sample members were born between 1 September 1989 and 31 August 1990. The sample design considered schools the primary sampling unit; among state-funded schools, deprived schools were over-sampled by 50%. The deprived schools were in the first quartile of free school meal recipients (i.e. pupils from a disadvantaged background are offered free school meals according to UK government policy) based on the Pupil Level Annual Schools Census. The school and pupil selection approach ensured that pupils had an equal probability of selection within a deprivation band (i.e. those in the first quartile of free school meal recipients) and ethnic group.

**Figure 1.**
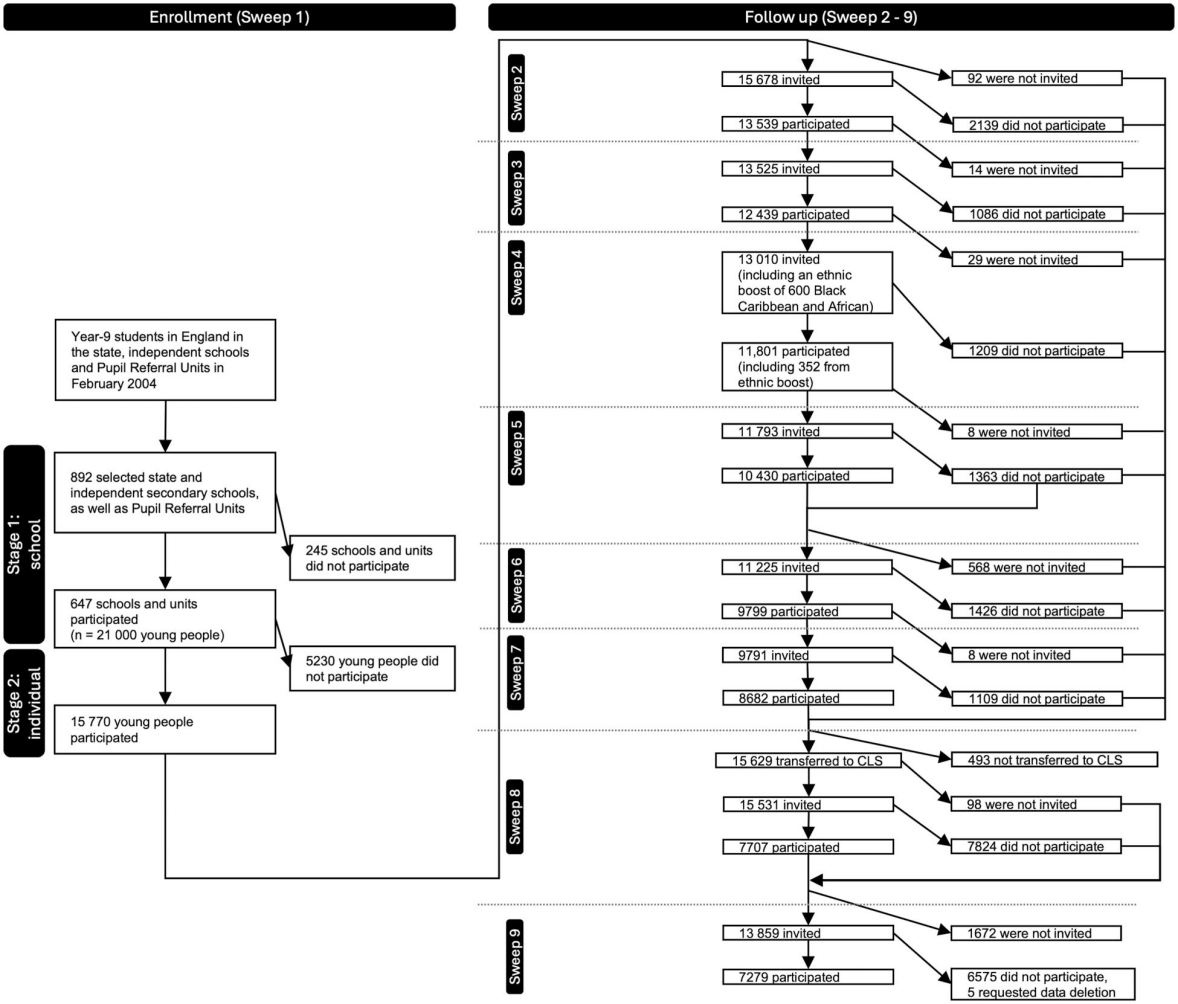
The enrolment and participation process of Next Steps. Of note, although at Wave 6, a substantial proportion of non-respondents who were invited at Wave 5 were re-invited to take part

The total number of cohort members who have ever participated in the study is 16 122, including 352 Black Caribbean and Black African pupils who joined the study at Sweep 4 as part of an ethnic minority boost. In addition to the young person, a main and a second parent were identified for interview in each sweep up to and including Sweep 4; from Sweep 5 onwards, only the cohort member has been interviewed. Figure 1 shows the enrolment, participation and follow-up process. A detailed description of the sampling is available in the Sweeps 1 to 7 user guide.^3^

The sample design included the oversampling of ethnic minorities and people from low socio-economic family backgrounds.^3^Table 1 shows the distribution of ethnicity, gender and social class (based on National Statistics Socio-economic classification for the household reference person) in the Next Steps sample, next to equivalent distributions in the National Pupil Database (NPD) in 2004 and those of the 2001 Census. As expected, due to the sample design, the unweighted Next Steps Sweep 1 sample has a higher proportion of ethnic minorities than other sources. After applying the appropriate design weight, the ethnic, gender and National Statistics Socio-economic classification distribution for Next Steps more closely aligns with the Census and the NPD sample. The design weight was calculated based on the combination of school and pupil non-response weights, which is calibrated to the NPD population proportion—the design weight is available in the dataset, and so too are non-response weights, which can be used to restore the representativeness of the sample at subsequent sweeps. Of note, oversampling ensures that certain subgroups within a population are adequately represented, allowing for more detailed analysis.^5^ Weighting adjusts the results to represent the population by compensating for oversampling and non-response.^6^

**Table 1.** The comparison between the Next Steps cohort Sweep 1 sample, National Pupil Database in 2004 and Census 2001

	**National Pupil Database (2004)** ^a^	**Census (2001)** ^b^	**Next Steps (2004) Unweighted** ^c^	Next Steps (2004) Weighted
Ethnicity				
White	83.0%	87.4%	67.0% (10 555)	87.7%
Bangladesh	1.0%	1.0%	4.7% (743)	0.9%
Pakistan	2.3%	2.2%	6.1% (963)	2.3%
Indian	2.3%	2.4%	6.5% (1019)	2.5%
African	1.8%	1.2%	4.0% (624)	1.1%
Caribbean	1.4%	1.2%	3.8% (596)	1.1%
Mixed	2.2%	2.8%	5.2% (815)	2.6%
Other	2.5%	0.9%	2.7% (429)	1.8%
Not obtained	3.5%	0%	0.2% (26)	2.1%
Ethnic minority, total	13.5%	12.6%	33% (5189)	12.3%
Sex				
Male	51.0%	51.2%	50.9% (7852)	51.5%
Female	49.0%	48.8%	49.1% (7579)	48.5%
National Statistics Socio-economic classification				
Higher managerial, administrative & professional	–	15.3%	10.3% (1619)	12.3%
Lower managerial, administrative & professional	–	26.2%	20.7% (3266)	22.7%
Intermediate	–	8.6%	6.5% (1018)	6.5%
Small employers and own account workers	–	10.2%	11.1% (1755)	11%
Lower supervisory and technical	–	10.3%	10% (1576)	10.4%
Semi-routine	–	10.7%	11.9% (1881)	11.6%
Routine	–	10.1%	10.7% (1687)	10.4%
Never worked and long-term unemployed	–	3.2%	7% (1100)	4.4%
Not classified	–	5.4%	11.8% (1868)	10.6%

a The proportion of National Pupil Database is retrieved from the Next Steps user guide.^3^ This is on the year group of the Next Steps sample.^3^

b The census data shown are on the age 10 to 14 population in England.^4^

c The percentage of each category in the Next Steps sample is presented with the number of the category.

d The National Statistics Socio-economic classification information in the 2001 census among the household reference persons aged 25–54 to compare with cohort members’ household reference persons. The ages were selected because more than 90% of the parents’ ages fall within this range.^28^

Among other studies, CLS is also home to three other national cohort studies born between 1958 and 2002: the National Child Development Study^7^ (NCDS, born in 1958), the British Cohort Study^8^^,^^9^ (BCS70, born in 1970) and the Millennium Cohort Study^10^ (MCS, born in 2000–02). Next Steps fills the gap between BCS70 and MCS cohort studies, representing the missing ‘millennial’ generation, enabling cross-cohort analyses which explore changes over time. Furthermore, the age of Next Steps participants is broadly similar to that in the Avon Longitudinal Study of Parents and Children,^11^ and Twins Early Development Study.^12^ This offers potential for wider pooled analyses or cross-cohort comparisons.

## How often have they been followed up?

For the first seven sweeps conducted annually (2004–10), only those who had participated in the previous sweep were invited to take part in the subsequent sweep. At Sweep 8, to maximize the achieved sample size, CLS invited all those who had ever participated in Next Steps (other than those who had opted out of their contact details being transferred from the Department for Education to CLS). At Sweep 9, all previous participants were again invited unless they had opted out of further follow-up, died, were in prison or on probation or had become permanently untraced.

The data for the first four sweeps of the study were collected via face-to-face interviewing and included interviews with cohort members’ parents to understand the family environment. From Sweep 5 onwards, interviewing involved the young person only and used a sequential mixed-mode approach. Cohort members could complete the interview online, over the telephone, by video (in Sweep 9) or face-to-face.

In addition to the main sweeps, three COVID-19 online surveys were conducted between 2020 and 2021. These surveys were simultaneously completed by participants in the three other CLS-housed cohorts (i.e. NCDS, BCS70 and MCS) and the National Survey of Health and Development^13^ (born in 1946), enabling cross-cohort analyses to understand the impact on individuals’ lives during the pandemic.^9^^,^^14^ The three surveys took place in May 2020, October 2020 and February 2021. In March 2021, study members were asked to take a COVID-19 antibody test.^15^

Table 2 shows the issued sample, the achieved sample and the response rate for each data collection sweep.

**Table 2.** The age, date of contact, sample sizes and response rate of each Next Steps cohort sweep between 2004 and 2023

Sweep	Age	School year	Date of contact	Invited	Participated	Response rate^a^
1	14	9	30 Mar–18 Oct 2004	21 000	15 770	74%
2	15	10	18 Apr–18 Sep 2005	15 678	13 539	86%
3	16	11	21 Apr–28 Sep 2006	13 525	12 439	92%
4	17	12	12 Jun–14 Oct 2007	12 468	11 449	92%
4 boost				600	352	59%
5	18	13	3 Jun–28 Oct 2008	11 793	10 430	88%
6	19		12 May–14 Oct 2009	11 225	9799	87%
7	20		18 May–12 Oct 2010	9791	8682	90%
8	25		20 Aug 2015–25 Sep 2016	15 531	7707	50%
COVID1	30		4–30 May 2020	9380	1907	20%
COVID2	30		10 Sep–16 Oct 2020	11 529	3664	32%
COVID3	31		1 Feb–21 Mar 2021	12 349	4239	34%
COVID_Antibody_	31		Mar 2021	4826	1267	26%
9	32		25 Apr 2022–24 Sep 2023	13 859	7279	53%

a The response rate in this table refers to the ratio of the number of responses received to the number of invitations issued. We do not use the baseline to calculate the response rate for the following reasons: (i) the issued invitations vary each time, especially for Sweeps 2–7, which only invite those who participated in the previous wave; (ii) during Sweep 4, we added 352 participants, bringing the total number of cohort members to 16 12;, and (iii) additionally, after Sweep 7, when transitioning to CLS, some cohort members chose to permanently opt out of the study, adjusting the total number to 15 629.

As noted in Table 2, the response rates varied across sweeps, and missingness was patterned. Silverwood *et al.*^16^ explored patterns or non-response for Next Steps. They took over 800 variables collected in Sweeps 1–7 and used a systematic, data-driven approach to identify important predictors of participation in Sweep 8, such as personal characteristics, behaviour at school, activities out of school, socioeconomic status, health (physical and mental) and practicalities around contact and survey completion. This paper informs the ongoing missing data strategy to ensure the representativeness of analysis of data subject to patterned non-response. More information on handling missing data is available on the CLS website [https://cls.ucl.ac.uk/data-access-training/handling-missing-data/].

## What has been measured?

The focus of this cohort is multidisciplinary, with rich information about individuals’ lived experiences and outcomes. As seen in Table 3, Next Steps covers various topics, including household composition, partnerships and information on current partners, children of cohort members, housing, current activities, including employment histories and occupations, household finances, general health and identity. Questions on some more sensitive topics, such as mental health and wellbeing, alcohol and drug use, sex, gender and sexual orientation, domestic violence, and adverse childhood experiences, were self-completed during interviewer-assisted interviews. Sweep 9 included a cognitive assessment task (the Backward Digit Span), which measures working memory. In Sweep 9, participants were asked to provide a saliva sample from which DNA would be extracted for genetic research; 57% of eligible respondents (i.e. 3591 out of 6352) agreed to provide saliva samples and were provided a kit to do so. A total of 48% (i.e. 1733 out of 3591) returned a sample for DNA extraction. After quality control, 1568 genotype data are available. More information on genomics data is available on this website [https://cls-genetics.github.io/docs/Next_steps.html].

**Table 3.** The measured variables from Next Steps cohort Sweeps 1 to 9

Theme and topics	1	2	3	4	5	6	7	8	9
Background									
Date of birth, gender, ethnicity, language and religion	⊛	⊚	⊚	⊚	⊚	⊚	⊚	⊚	⊚
Household composition ans relationships									
Household composition	⊕	⊕	⊕	⊕			⊚	⊚	⊚
Housing tenure	⊕	⊕	⊕	⊕	⊚	⊚	⊚	⊚	⊚
Relations between cohort member and parents	⊕	⊕	⊕	*					
Relationships and sexuality						⊚	⊚	⊚	⊚
Social connection		⊚						⊚	⊚
Childcare and caring responsibilities				⊚	⊚	⊚	⊚	⊚	⊚
Household responsibilities and resources	⊛	⊛	⊛	⊕					
Sibling information	⊕	⊕		*					
Education									
Subjects/qualifications being studied for/acquired		⊚	⊚	⊚	⊚	⊚	⊚	⊚	⊚
Higher education or future plans	⊛	⊚	⊚	⊚*	⊚	⊚	⊚		
Higher education experience					⊚	⊚	⊚		⊚
Attitudes to school/teachers	⊚	⊚	⊚	⊚		⊚			
Cohort member’s special educational needs	⊕	⊕	⊕	*					
School history	⊕	⊕							⊚
Finances for education				⊚	⊚				⊚
Employment and resources									
Current activities and activity history			⊚	⊚	⊚	⊚	⊚	⊚	⊚
Occupation types				⊚	⊚	⊚	⊚	⊚	⊚
Jobs and training			⊚	⊚	⊚	⊚	⊚	⊚	⊚
Apprenticeships		⊚	⊚	⊚	⊚	⊚	⊚		
Reasons for not being in education, employment or training				⊚	⊚	⊚	⊚		⊚
Income and benefits			⊚	⊚	⊚	⊚	⊚	⊚	⊚
Attitudes to work						⊚	⊚		⊚
Health and wellbeing									
Cohort member's health and disability	⊕	⊛	⊚	⊚		⊚	⊚	⊚	⊚
Pregnancy and fertility experience									⊚
Mental and emotional health		⊚		⊚				⊚	⊚
Sport frequency	⊚	⊚	⊚	⊚		⊚	⊚	⊚	⊚
Diet								⊚	⊚
Substance use	⊚	⊚	⊚	⊚		⊚	⊚	⊚	⊚
Coronavirus experience									⊚
Saliva sample									⊚
Height and weight								⊚	⊚
Backward digit sequence test									⊚
Identity and participation									
Importance of ethnicity, nationality as part of identity									⊚
Gender diverse or sexual identity						⊚	⊚	⊚	⊚
Risk factors (absences, truancy, police contact, bullying, domestic violence)	⊛	⊛	⊛	⊚		⊚	⊚	⊚	⊚
Social attitudes and participation (e.g. social equality, voting behaviour/intension)					⊚		⊚		⊚
Locus of control		⊚		⊚			⊚	⊚	
Use of leisure time	⊚	⊚		⊚				⊚	
Grit (e.g. passion and perseverance)									⊚
Parent information									
Demographics (e.g. gender, ethnicity, place of birth)	⊕	⊕		⊕*	⊚				⊚
Health and disability	⊕	⊕		⊕*					
Parents' employment/activity history	⊕	⊕	⊛	⊕					
Employment training and earnings	⊕	⊕	⊕	⊕	⊚				
Qualifications and education	⊕	⊕		*					
Benefits and tax credits	⊕	⊕		⊕	⊚				
Relationship history	⊕	⊕		⊕*					
Parental expectations and aspirations	⊕	⊕	⊕	*					
Religion	⊕								
Partner's information									
Education								⊚	⊚
Demographics (e.g. gender, age, ethnicity)								⊚	⊚
Employment								⊚	⊚
Work changes due to COVID19 pandemic									⊚
Income and benefits								⊚	⊚
Education data consent									
Student Loans Company								⊚	⊚
Universities and Colleges Admission Service								⊚	⊚
Department for Education (including National Pupil Database and Individual Learner Record)	⊚						⊚	⊚	⊚
Department for Business, Innovation and Skills (including Higher Education Statistics Agency)							⊚	⊚	⊚
Economic data consent									
Department of Work and Pensions				⊚	⊚	⊚	⊚	⊚	⊚
HM Revenue and Customs								⊚	⊚
Criminal data consent									
Ministry of Justice								⊚	⊚
Heath data consent									
National Health Service								⊚	⊚

⊕ , parents;

⊚ , cohort member;

⊛ , cohort member and parents;

* , boost; in the Next Steps cohort (2004 to 2023)

Parental information, including parental education and family benefits, was collected between Sweeps 1 and 4. Table 3 summarizes some of the key measured variables.

Additionally, the survey data have been enhanced through linkage to administrative, geospatial and environmental data. At the initial recruitment stage, all parents consented for NPD data to be linked to the study data. NPD provides information, including pupils’ school attainment at Key Stages 2–4 (school years 3–11), free school meal eligibility and special educational needs. In Sweeps 8^17^ and 9, permission was additionally sought to add information from the administrative sources in Table 3. Next Steps data linked to NPD, Individual Learner Record, Student Loans Company data and Hospital Episode Statistics are currently available,^17^^,^^18^ and other data linkages are underway. The linked geospatial and environmental data include proximity to local amenities, green space availability, air pollution and local crime rates.

The COVID-19 surveys collected information including COVID-19 infection and vaccination, time use, family and household, housing, finance, pre- and current COVID-19 employment, health behaviours, social support, mental health and life events, see Table 4. More details can be found in the COVID-19 survey user guide.^14^

**Table 4:** Summary of Next Steps cohort COVID-19 surveys (2020–21)

Theme and topics	1	2	3
Physical health			
COVID-19 infection and symptoms	⊚	⊚	⊚
Long COVID-19 symptoms			⊚
Extent of compliance with social distancing guidelines	⊚		⊚
Vaccination			⊚
Self-rated general health	⊚	⊚	⊚
Long-standing health conditions	⊚	⊚	⊚
Disruption of medical appointments or treatments	⊚	⊚	⊚
Family and household			
Household grid	⊚	⊚	⊚
Changes in household composition	⊚	⊚	⊚
Relationship satisfaction/conflict	⊚	⊚	⊚
Household care needs and receipt of care	⊚	⊚	⊚
Housing			
Number of rooms in house	⊚	⊚	⊚
Tenure	⊚	⊚	⊚
Finance			
Subjective assessment of how managing financially pre and post outbreak	⊚	⊚	⊚
Food security, use of food banks	⊚		
New claims for benefits since outbreak	⊚	⊚	⊚
Giving/receiving financial help		⊚	⊚
Employment			
Pre-COVID and current activity (occupation, hours, contract type)	⊚	⊚	⊚
Current work location	⊚	⊚	⊚
Job satisfaction		⊚	⊚
Home working satisfaction		⊚	⊚
Education			
Subject, name of institute, course length, current year of study	⊚	⊚	⊚
Disruption of teaching/online learning	⊚	⊚	⊚
Satisfaction with learning provision/academic progress	⊚	⊚	⊚
Health behaviour			
Substance use	⊚	⊚	⊚
Physical activity, sleep, diet	⊚	⊚	⊚
Weight	⊚	⊚	⊚
Screen time			⊚
Mental and social health			
Contact with friends and family	⊚	⊚	⊚
Engagement in online activities	⊚	⊚	⊚
Social support	⊚	⊚	⊚
Loneliness	⊚	⊚	⊚
Life satisfaction	⊚	⊚	⊚
Mental ill health or anxiety	⊚	⊚	⊚
Attitude			
Optimism, risk, patience, trust	⊚	⊚	⊚
Trust in government and political leaders	⊚	⊚	⊚
Children (for children aged 4–18 living in household)			
Education (enrolment, school year, home learning, learning resources, impact of pandemic on progress)		⊚	⊚

## What has it found?

A wide range of research has been undertaken using Next Steps data, totalling over 300 peer-reviewed publications, as documented in the CLS bibliography [www.cls.ioe.ac.uk/bibliography]. We highlight three featured research themes: mental health, health behaviours and social mobility.

### Mental health

Previous studies used Next Steps to explore the associations between mental health, education and economic activities at different life stages. For example, Symonds *et al*.^19^ found that young people’s decisions after age 16, such as not being in employment, education or training, or transiting to vocational education or full-time employment, were associated with changes in mental health and functioning between ages 15 and 17. In addition, although prior studies indicated the benefits of private schooling regarding subsequent labour market outcomes,^20^^,^^21^ Henderson *et al*.^22^ found that among the Next Steps cohort, there was no evidence to support a positive association of private schooling on mental health and life satisfaction.

### Health behaviours

Next Steps also provides information on health behaviours, including smoking, substance usage, exercise and sex.^12^ One study explored substance use, including cigarette, alcohol and cannabis, and found that higher academic attainment at age 11 was associated with a reduction in the likelihood of cigarette use but an increase in the likelihood of alcohol and cannabis use in adolescence and early adulthood.^23^ Mendolia and Walker^24^ found that adolescents aged 15–16, who had high external locus of control, low self-esteem and low work ethic, were more likely to be involved in risky health behaviour, such as alcohol/drug use, and unsafe sexual activity.

### Social mobility

Regarding social mobility, Chatzitheochari *et al*.^25^ found that disabled young people had lower labour participation and were less likely to have upward intergenerational social mobility than their non-disabled peers. Other research has explored the association between parental education and adolescents’ education decisions after compulsory education. For example, Henderson *et al*.^26^ found that students who were the first person going to a university in the family were more likely to enrol on degrees with higher earning potential (such as Law, Economics and Management), were more likely to drop out from university and were more likely to study at a non-Russell Group university compared with those with university-educated parents.^26^

## What are the main strengths and weaknesses?

Like all longitudinal studies, this cohort faces selective attrition which can be addressed by the many techniques described in the CLS Missing Data strategy, with the aim to restore the sample's representativeness. The sample size of the latest sweep of Next Steps (>7000) is sufficient to continue to follow across the life course, enabling exploration or longer-range outcomes. As noted, the sampling design of Next Steps over-sampled those from deprived schools and ethnic minority groups at baseline, which has ensured adequate sample sizes for sub-group analyses. This has benefited research on inequalities in health, education and social mobility.

Next Steps provides many valuable insights; however, there are some limitations with respect to generalizability—the extent to which any findings can be applied to other contexts or populations. Next Steps is representative of people who were enrolled in an English school in 2004 and who are alive and not in prison or on probation in 2022–23. However, the large sample size of Next Steps, oversampling of particular groups, and ability to replicate findings with other cohort studies who are similarly aged, can help offer some assurance of reliable results.

The in-depth education and life experience information during the transition between adolescence and adulthood—a critical period for establishing and consolidating a healthy lifestyle—can help to establish longer-term pathways to good health. Additionally, the broadened scope of Next Steps in adulthood extended to diverse aspects, including fertility intentions, DNA, working memory, cost of living, and employment, as well as the breadth of administrative data linkage consents, will facilitate many forms of policy-relevant research on the intersection of health, socioeconomic conditions and environment.

The study's intergenerational nature allows researchers to explore how inequalities are transmitted throughout families. Next Steps enables cross-cohort analyses, as it fills the gap between BCS70 and MCS, capturing the experiences of the millennial generation which faced unique challenges.

Taken together, the rich information provided by Next Steps facilitates research which can be directly translated into strategies to improve the quality of lives in a timely way.

## Can I get hold of the data? Where can I find out more?

Next Steps is managed by CLS, Institute of Education, University College London, under the direction of Professor Morag Henderson. Detailed documentation, research and training materials are available on the Next Steps website [https://cls.ucl.ac.uk/cls-studies/next-steps/]. Next Steps data, including the latest sweep (Sweep 9), are available,^27^ free of charge, to researchers and can be accessed through public repositories, primarily the UK Data Service (UKDS) [https://beta.ukdataservice.ac.uk/datacatalogue/series/series?id=2000030] and also the UK Longitudinal Linkage Collaboration [https://ukllc.ac.uk/datasets]. Some datasets, such as linked administrative or sensitive data, are only available via the UK Data Service Secure Lab, which requires additional training. More information can be found in UKDS website. Data not publicly available via these public repositories, and proposals for novel data linkages and data enhancements, can be requested directly from CLS [https://cls.ucl.ac.uk/data-access-training/data-access/]. The Next Steps website [https://cls.ucl.ac.uk/cls-studies/next-steps/next-steps-age-32-sweep/] has more information.

## Ethics approval

The Department for Education managed ethical approval for Sweeps 1–7 (ages 14–20). CLS received ethical approval for Sweep 8 (age 25) from the NHS Research Ethics Committee (REC), (REC Reference 14/LO/0096), and for Sweep 9 (age 32) from the NHS REC (REC Reference 22/EE/0052).^28^

## Data Availability

See ‘Can I get hold of the data?’ above.
